# Development and experimental verification of a genome-scale metabolic model for *Corynebacterium glutamicum*

**DOI:** 10.1186/1475-2859-8-43

**Published:** 2009-08-03

**Authors:** Yohei Shinfuku, Natee Sorpitiporn, Masahiro Sono, Chikara Furusawa, Takashi Hirasawa, Hiroshi Shimizu

**Affiliations:** 1 Department of Bioinformatic Engineering, Graduate School of Information Science and Technology, Osaka University, 1-5 Yamadaoka, Suita, Osaka 565-0871, Japan; 2 ERATO Complex Systems Biology Project, JST, 1-5 Yamadaoka, Suita, Osaka 565-0871, Japan

## Abstract

**Background:**

*In silico *genome-scale metabolic models enable the analysis of the characteristics of metabolic systems of organisms. In this study, we reconstructed a genome-scale metabolic model of *Corynebacterium glutamicum *on the basis of genome sequence annotation and physiological data. The metabolic characteristics were analyzed using flux balance analysis (FBA), and the results of FBA were validated using data from culture experiments performed at different oxygen uptake rates.

**Results:**

The reconstructed genome-scale metabolic model of *C. glutamicum *contains 502 reactions and 423 metabolites. We collected the reactions and biomass components from the database and literatures, and made the model available for the flux balance analysis by filling gaps in the reaction networks and removing inadequate loop reactions. Using the framework of FBA and our genome-scale metabolic model, we first simulated the changes in the metabolic flux profiles that occur on changing the oxygen uptake rate. The predicted production yields of carbon dioxide and organic acids agreed well with the experimental data. The metabolic profiles of amino acid production phases were also investigated. A comprehensive gene deletion study was performed in which the effects of gene deletions on metabolic fluxes were simulated; this helped in the identification of several genes whose deletion resulted in an improvement in organic acid production.

**Conclusion:**

The genome-scale metabolic model provides useful information for the evaluation of the metabolic capabilities and prediction of the metabolic characteristics of *C. glutamicum*. This can form a basis for the *in silico *design of *C. glutamicum *metabolic networks for improved bioproduction of desirable metabolites.

## Background

A coryneform bacterium, *Corynebacterium glutamicum *is a facultatively aerobic, gram-positive bacterium that can grow on various sugars or organic acids [1,2]. This organism can produce various amino acids such as glutamate [1,2] and lysine [3] with high efficiency, and is thus widely used for the large-scale production of amino acids [4,5]. Furthermore, the production of ethanol and organic acids such as lactate and succinate by growing *C. glutamicum *under oxygen deprivation conditions has recently been proposed [6,7]. Owing to its importance for bioproduction, *C. glutamicum *has been chosen as one of the effective hosts for metabolic engineering purposes [8-10]. Thus, the construction and exploration of appropriate *in silico *metabolic models, which help predict the cellular behavior and production of useful chemicals, are highly desired.

Recently, on the basis of whole-genome information, the genome-scale metabolic networks of cells have been reconstructed and applied to metabolic flux balance analysis (FBA) [11,12] for many organisms, including representatives of each of the 3 major domains of life, namely, archaea [13], bacteria [14-17], and eukarya [18-20]. FBA is an analysis of metabolic flux profiles, in which a steady state of metabolic flux is assumed and the profile of metabolic fluxes is calculated by optimizing an objective function using linear programming. Although genome-scale metabolic models cannot compute the detailed kinetic dynamics of metabolic reactions in a cell, they enable the description of the range of possible metabolic states on the basis of the constraints defined by the stoichiometry of metabolic reactions and transport steps at a steady state. Furthermore, we can obtain a solution, i.e., a set of all metabolic fluxes, which maximizes an objective function using linear programming. The biomass production rate is generally adopted as the objective function. It has been shown that the metabolic profiles calculated by the maximization of biomass production can describe those obtained experimentally in many organisms and environmental conditions, suggesting that organisms can maximize their growth rate by adaptation and evolution [21,22]. Using the appropriate genome-scale metabolic network and an objective function to be maximized, FBA can be used to predict the relationship among the genotype, environmental conditions, and product yields at the steady state; this data can be utilized for the improvement of the microbial production [23,24].

In this study, we present the reconstruction of a genome-scale metabolic model of *C. glutamicum*. Metabolic reactions and other parameters for biomass were collected using databases and literatures. After reconstruction of the genome-scale metabolic model, we performed FBA simulations with the maximization of biomass production and evaluated the results of the simulations by using experimental data of *C. glutamicum *cultures grown at different oxygen uptake rates (OURs). Our results revealed that the production rates of biomass and organic acids predicted using our model agreed well with the experimental rates; this result suggests that this model well represents the intracellular metabolic profiles of *C. glutamicum*. It should be noted that the representation of such changes in the metabolic profiles that occur on changing the oxygen uptake is difficult for another genome-scale model of *C. glutamicum *reported recently [25]. Furthermore, by using the model proposed in this paper, we performed comprehensive simulations of gene deletions in order to identify candidate genes for genetic modification(s) to improve the productivity of organic acids by *C. glutamicum*.

## Materials and methods

### Modeling and Simulations

#### Network reconstruction

The known metabolic reactions in the *C. glutamicum *metabolic network were collected by a search of public databases and scientific publications. The genome-scale metabolic network was based on the pathways in the BioCyc database collection [26] for *C. glutamicum*. We also referred to the information on *C. glutamicum *in the Kyoto Encyclopedia of Genes and Genomes database (KEGG; ). In general, the genome-scale model constructed using only public databases contains incorrect and insufficient metabolic pathways owing to the incompleteness of database information. The most frequently observed incorrectness was missing enzymes in metabolic pathways. Thus, the resulting network was then subjected to the gap-filling process to allow biomass formation. For gap filling, we referred to published cell-specific data available in the literatures, such as Ref. [27,28] and references threin. For example, the mycolic acid and arabinogalactan synthetic pathways are constructed on the basis of Refs. [29,30].

#### Determination of biomass composition

The reaction "biomass synthesis" is a hypothetical metabolite in the metabolic network, which represents the requirement of precursors and coenzymes for the biomass formation. Biomass synthesis consists of a linear combination of 43 components, including amino acids, DNA, RNA, lipids, and cell envelope components. In our model, the biomass composition was determined from various reported data. The demands of precursors such as pyruvate, acetyl-CoA, and oxaloacetate for biomass production were based on the data in Ref. [27]. For the macromolecular composition, we referred to the data in Ref. [31]; the total biomass is composed of 52% protein, 5% RNA, 1% DNA, 13% lipid, 19% cell wall components, and 10% other components. The *C. glutamicum *cells have a characteristic cell membrane termed as MAPc [32], which consists of the polysaccharides peptidoglycan and arabinogalactan as well as mycolic acids. Since the MAPc biosynthesis pathway is quite complex and some metabolic reactions in MAPc synthesis have not been characterized in detail, we described this pathway by some lumped reactions. The biomass composition of MAPc was determined to satisfy the macromolecular composition and the precursor demand described above. The biomass compositions of nucleic acids were calculated on the basis of the genomic sequence [33]; the composition ratios of DNA and RNA to the total biomass are 5% and 1%, respectively [31]. The biomass synthesis reactions and its composition are shown in additional file 1. For all simulations presented here, the composition of every component was fixed independent of the environmental conditions. The energy requirement for biomass production was set to 41.26 mmol of ATP per 1 g biomass on the basis of the data in Ref.[34].

#### *In silico *computation: FBA

Metabolic fluxes of the *C. glutamicum *metabolic network were calculated by using flux balance analysis (FBA), in which constrains are imposed by the stoichiometry in a metabolic network [11]. A pseudo-steady state is generally assumed, i.e., the net sum of all production and consumption fluxes for each internal metabolite is set to zero. This assumption results in a feasible space that is a convex set in the N-dimensional space of metabolic fluxes (where N stands for the total number of fluxes). In FBA, a particular objective function, written as a linear combination of fluxes, can be used to calculate the optimal solution at 1 corner in the feasible flux space. Using the matrix notation, this problem can be stated as follows:







where **S **is the stoichiometric matrix representing the stoichiometry of metabolic reactions in the network and ***v ***is a vector of all metabolic fluxes. ***v***_min _and ***v***_max _indicate the minimum and maximum constraints on the fluxes and are used to define the constraints for maximal enzymatic rate, irreversibility of reaction, or constant uptake from the environment. ***c***^T ^is a vector representing the objective function to be maximized, as a linear combination of metabolic fluxes. In general, the biomass production rate mentioned above is used as the objective function to be maximized. In this study, we followed this method to calculate the metabolic flux profile under the assumption that organisms have been evolved toward growth maximization. For all simulations in this paper, glucose was chosen as the sole carbon source and the following external metabolites were allowed to freely transport through the cell membrane: CO_2_, H_2_O, SO_3_, NH_3_, and PO_4_. All calculations, including the linear programming problems, were performed using the commercially available software Lindo (Lindo Systems, Inc.) and Matlab (Mathworks, Inc.).

#### Strain and medium

*C. glutamicum *strain ATCC 13032 was used in the culture experiments. The composition of the synthetic medium used for the preculture of the microorganism was the same as that employed in our previous study [35] (per liter of deionized water): 40 g of glucose, 30 g of (NH_4_)_2_SO_4_, 3.0 g of Na_2_HPO_4_, 6.0 g of KH_2_PO_4_, 2.0 g of NaCl, 84 mg of CaCl_2_, 3.9 mg of FeCl_3_, 0.9 mg of ZnSO_4_.7H_2_O, 0.1 mg of (NH_4_)_6_Mo_7_O_21_.4H_2_O, 0.3 mg of Na_2_B_4_O_7_.10H_2_O, 0.4 mg of MgSO_4_.7H_2_O, 40 mg of FeSO_4_.7H_2_O, 500 μg of vitamin B1·HCl, 0.1 g of EDTA, and 10 μg of biotin. The medium composition for the main culture was the same as that for the preculture except the initial glucose concentration was changed to 80 g/L for batch cultivation and 20 g/L for continuous cultivation.

#### Culture conditions

**Figure 1:**
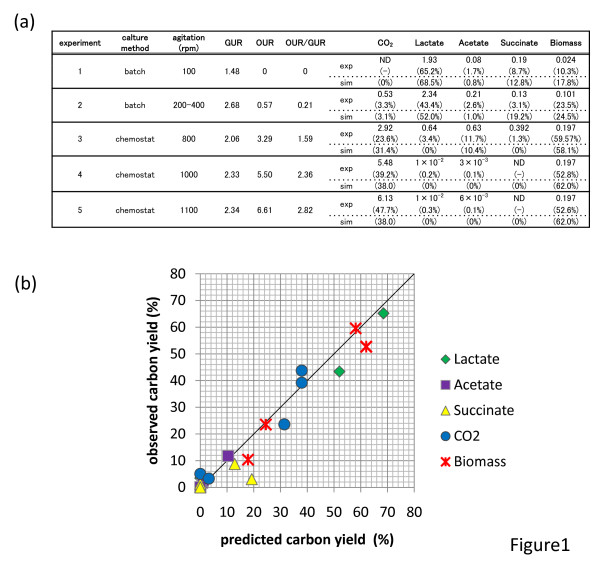
**Changes in the yields of organic acids, biomass, and carbon dioxide on changing OUR/GUR ratio**. (a) Summary of experimental results and predictions by FBA simulations. The unit of GUR, OUR, and production rates of CO_2_, lactate, acetate, succinate, and biomass are mmol/gDW/h. The values in parentheses represent carbon yields. Simulation results which were obtained by using the same GUR and OUR are also presented. (b) A scatter plot of carbon yield. The x-axis corresponds to the result of FBA simulation, while the y-axis show the experimentally observed carbon yield. The carbon yields in the 5 sets of experimental and simulation results are presented. The line corresponding to y = x is also included.

For microaerobic culture conditions (experiments 1 and 2 shown in Fig. 1(a)), the batch cultivations of *C. glutamicum *were carried out using a 500-mL jar fermenter (model BMJ-P, Able, Japan) with a liquid working volume of 200 mL. In the batch cultures, the cells were first allowed to grow aerobically at a high aeration rate (1 vvm; volume of air per volume of medium per min) and high agitation speed (800 rpm) until the cell concentration, as measured by the optical density at 660 nm (OD_660_), reached 5~15. Then, for experiment 1, the culture conditions were changed to no aeration and gentle agitation (100 rpm) and for experiment 2, the aeration rate was altered to 0.5 vvm and the OUR was maintained at a constant value (0.5 mmol/gDW/h) by changing the agitation speed. For the aerobic cultures (experiments 3, 4, and 5), chemostat cultivations were carried out using a 500-mL jar fermenter with a liquid working volume of 200 mL. The aeration rate was fixed at 1 vvm and the dilution rate, at 0.2 h^-1 ^for all chemostat culture experiments. In order to change the OUR in these chemostat cultures, the agitation speed was set to 800, 1000, and 1100 rpm for experiments 3, 4, and 5, respectively.

All culture experiments were performed at 31.5°C, and the pH was maintained at 7.2 by the automatic addition of 25% ammonium solution using an autoclavable pH probe (Fermprobe F-635; Broadley-James Corporation, Irvine, CA) and a pH controller (DT-1023; Able Corporation, Japan).

### Analytical Methods

Cell growth was monitored by measuring the OD_660_. The dry cell weight (DCW) was calculated using the measured OD_660 _according to the following formula:



The culture supernatant was used for measuring the concentration of glucose and organic acids. Glucose was measured using an enzymatic assay kit, the Glucose CII test Wako (Wako Pure Chemicals, Inc., Japan) according to the manufacturer's protocol. Organic acids were quantified using a HPLC system, Hitachi L-6200 equipped with a L-4000H UV detector (210 nm; HITACHI, Japan). Samples were eluted with 0.75 mM H_2_SO_4 _in an ion-exclusion column TSKgel Oapak-P (Tosho, Japan) at 40°C. The flow rate was set to 0.8 ml/min. The concentration of dissolved oxygen (DO) in the culture was monitored by using an oxygen electrode (DKK-Toa Corporation, Japan), and the concentrations of exhaust oxygen and carbon dioxide were monitored by using an exhaust gas analyzer (Model DEX-1562-1; Able Corporation, Japan).

## Results and discussions

### Reconstruction of the metabolic network

We reconstructed a genome-scale metabolic network for *C. glutamicum *ATCC 13032, whose genomic DNA sequence was determined by 2 independent research groups [33,36]; the metabolic network consists of 277 genes, 502 metabolic reactions, and 423 metabolites. The entire reaction data set is provided in additional file 1. A total of 428 reactions from the BioCyc and KEGG database collections were included in the model, while the remaining 74 reactions were added on the basis of previously published studies of each pathway [27,28]. The basic characteristics of the reconstructed metabolic network are presented in Table 1, in which the genomic features of *C. glutamicum *were obtained from Ref.[33]. From the entire set of reactions, 470 correspond to intracellular reactions, while 32 are fluxes for transport through the membrane. The model includes 391 intracellular metabolites and 32 extracellular metabolites. The functional classification of the 502 metabolic reactions in the reconstructed model is summarized in Table 2, in which the reaction "Biomass synthesis" indicates the hypothetical reaction to synthesize biomass. Transport processes were added to the model by reference to the BioCyc database collection and transport classification database (TCDB; ) and by inference from physiological considerations and genome annotations [27].

**Table 1:** Genomic features and characteristics of a reconstructed metabolic model of *C. glutamicum *ATCC 13032.

Feature	Property
***Genome characteristics***	
Genome length	3282708 bp
G+C content	53.80%
No. of open reading frames (ORFs)	3432
Total coding sequences (CDS)	3002
CDS encoding annotated proteins	2489
	
***In silico metabolic networks***	
No. of genes included	277
No. of associated reactions	428
No. of other reactions	74
No. of metabolites	423
No. of internal fluxes	470
No. of exchange fluxes	32

The genomic features of *C. glutamicum *ATCC 13032 shown here were reported by Kalinowski et al. [33].

**Table 2:** Functional classification of metabolic reactions in a *C. glutamicum *genome-scale model.

**Carbohydrate metabolism**	**45**	**Metabolism of complex carbohydrate**	**66**
glycolysis/gluconeogenesis	18	arabinogalactan biosynthesis	2
TCA cycle	17	dTDP-rhamnose biosynthesis	5
pentose phosphate pathway	8	D-lactate metabolism	5
Entner-Doudoroff pathway	2	GDP-mannose metabolism	5
**Energy metabolism**	**17**	glycerol and glycerophosphodiester degradation	2
**Lipid metabolism**	**32**	mevalonate pathway	9
fatty acid biosynthesis	15	UDP-N-acetylgalactosamine biosynthesis	7
phospholipid biosynthesis	17	UDP-glucose conversion	3
**Nucleotide metabolism**	**84**	isopentenyl diphosphate biosynthesis	8
PRPP biosynthesis	1	glutathione redox reactions	4
purinebiosynthesis	29	myo-inositol biosynthesis	2
pyrimidine biosynthesis	23	Polysaccharide biosynthesis	1
nucleotide salvage pathway	31	peptidoglycan biosynthesis	**13**
**Amino acid metabolism**	**103**	**Metabolism of complex lipid**	13
glutamate biosynthesis	2	MAPc biosynthesis	1
glutamine biosynthesis	1	PIM2 biosynthesis	2
alanine biosynthesis	3	Mycolyl-ACP biosynthesis	1
valine biosynthesis	3	polyamine biosynthesis	7
aspartate biosynthesis	1	Corynomycolate biosynthesis	2
lysine biosynthesis	11	**Metabolism of cofactors and vitamins**	**67**
arginine biosynthesis	9	ATP maintenance	1
asparagine biosynthesis	2	coenzyme A biosynthesis	5
threonine biosynthesis	2	folate transformations	2
isoleucine biosynthesis	5	formylTHF biosynthesis	9
leucine biosynthesis	4	NAD biosynthesis	19
proline biosynthesis	5	O-antigen biosynthesis	1
serine biosynthesis	3	pantothenate biosynthesis	6
tyrosine biosynthesis	3	riboflavin and FMN and FAD biosynthesis	9
tryptophan biosynthesis	6	tetrahydrofolate biosynthesis	15
cysteine biosynthesis	2	**Metabolism of other amino acids**	**9**
phenylalanine biosynthesis	3	homoserine biosynthesis	1
glycine biosynthesis	1	chorismate biosynthesis	7
methionine biosynthesis	17	spermine biosynthesis	1
histidine biosynthesis	10	**Metabolism of sugars**	**14**
interconversion of arginine, ornithine and proline	10	trehalose biosynthesis	2
		starch biosynthesis	12
		**Transport pathway**	**31**
		**Biomass synthesis**	**1**
		**Exchange pathway**	**34**

The reaction "Biomass synthesis" indicates the hypothetical reaction to synthesize biomass, which represents the requirement of precursors and coenzymes for the biomass formation.

The reconstructed metabolic network of *C. glutamicum *has several characteristics that distinguish it from the networks of other microorganisms. The cell envelopes of coryneform bacteria and mycobacteria have a unique structure consisting of a covalently linked complex comprising mycolic acid, arabinogalactan, and peptidoglycan (MAPc) [32]. In order to represent the characteristics of cell envelope biosynthesis, we introduced metabolic reactions for MAPc biosynthesis into the model. The synthetic pathways and composition of MAPc were decided on the basis of previous studies [29,30] and the demand of MAPc for biomass production was considered to be consistent with the cell wall composition of *C. glutamicum*. In the central metabolic pathway, the reconstructed model of *C. glutamicum *has pyruvate carboxylase in the anaplerotic pathway and lacks pyruvate formate lyase. In addition, *C. glutamicum *has 2 pathways for the biosynthesis of diaminopimelate, which is a precursor for lysine biosynthesis. One pathway involves the direct conversion of Δ1-piperideine-2,6-dicarboxylate to diaminopimelate, which is catalyzed by diaminopimelate dehydrogenase; the other pathway involves an indirect conversion, which is catalyzed by 4 independent enzymes [37]. Additionally, *C. glutamicum *does not have glycine C-acetyltransferase, which is involved in the conversion of threonine to glycine. These distinguishing characteristics of the metabolic pathways are necessary to represent the flux profile of *C. glutamicum*.

### Verification of the *C. glutamicum *genome-scale model: Measuring metabolic profiles under various oxygen supply conditions

In order to verify the results of the FBA using our genome-scale model of *C. glutamicum*, we compared the growth and metabolic profiles obtained by FBA simulations with those obtained by experiments performed at various oxygen levels. Here, we focused on the OUR as a parameter to change the metabolic profiles of *C. glutamicum*; this is because OUR is known to alter the metabolic profile drastically and is a key factor for controlling the productivity of several materials by this microorganism, such as organic acids. We performed a series of experiments involving the culture of *C. glutamicum *ATCC 13032 at various oxygen levels. The results of these experiments are summarized in Fig. 1(a). For aerobic conditions, i.e., conditions with relatively high OURs, we used continuous cultures with a dilution rate of 0.2 h^-1^; in these cultures, the oxygen supply was changed by altering the agitation speed to 800, 1000, and 1100 rpm (experiments 3, 4, and 5 in Fig. 1(a)). Here, the steady state was defined as that CV of cell concentration measured by optical density (OD_660_) is less than 10% over 10 hours. The production rates of CO_2 _and organic acids were measured in the steady states of the continuous cultures. We also confirmed that, for GUR, OUR, and production rates of organic acids, the ranges of deviation from the means were less than 5% during the steady state. In microaerobic and oxygen anaerobic conditions, *C. glutamicum *exhibited low specific growth rate (e.g., less than 0.05 h^-1^), and maintaining a steady state in the continuous cultures was difficult. Thus, we used batch cultures in this study and altered the oxygen supply conditions during the culture. In the batch cultures, cells were first grown under aerobic conditions with aeration and a high agitation speed; during the exponential growth phase, nitrogen gas started to be supplied instead of air for maintaining the anaerobic condition (experiment 1) and the agitation speed was controlled to maintain a constant OUR for the microaerobic condition (experiment 2). After changing the oxygen supply conditions, the production rates of CO_2 _and organic acids were measured (the time series of cell concentration and organic acid concentrations in experiments 1 and 2 are presented in additional files 2 and 3). Here, we obtained GUR, OUR, and the production rate of organic acids by linear regression of the time series. To check the error of this regression, we also calculated the confidence intervals of the regression and found that the 95% confidence intervals are less than 10% from the mean. In experiment 1, data of OUR was set to zero since nitrogen gas was supplied to the fermentor instead of air. As shown in Fig. 1(a), the production rates of biomass, CO_2_, and organic acids depended on the oxygen supply conditions. The experimental data presented in Fig. 1(a) indicated that under the anaerobic and micro-aerobic conditions (experiment 1 and 2), the cells converted most of the glucose to organic acids as lactate and succinate. With an increase in OUR/GUR ratio, cells changed their metabolism to produce acetate (experiment 3), and a further increase in OUR/GUR ratio resulted metabolic shift to CO_2 _production phase in which the tricarboxylic acid (TCA) cycle was activated (experiment 4 and 5).

Using the experimental data summarized in Fig. 1(a), we evaluated the results of the FBA simulations of our genome-scale model. In these simulations, we used biomass maximization as the objective function of FBA. We calculated the production yields of organic acids, CO_2_, and biomass using experimentally observed OUR and GUR values. The predicted yields are presented in Fig. 1(a). Also, in Fig. 1(b), we show a scatter plot of carbon yields, in which the carbon yields in the 5 sets of experimental and simulation results are presented. In Fig. 1(b), x-axis shows the predicted yield by FBA simulation and y-axis represents experimentally observed ones. As shown in the Fig. 1(a) and 1(b), the predictions of our genome-scale model with maximization of the biomass yield agreed well with the experimentally obtained yields. For example, the FBA simulation predicted that 10% of carbon is secreted as acetate when OUR/GUR ~1.5, which is consistent with the experimental result. The most significant discrepancy between the experimental results and FBA simulation was in the succinate production yield under the micro-aerobic and anaerobic conditions. The FBA simulation with biomass production maximization predicted that 10~20% of carbon is secreted as succinate in that condition, and the experimental results revealed that around 5% of carbon was secreted as succinate. The possible cause of this discrepancy will be discussed in the next section.

**Figure 2:**
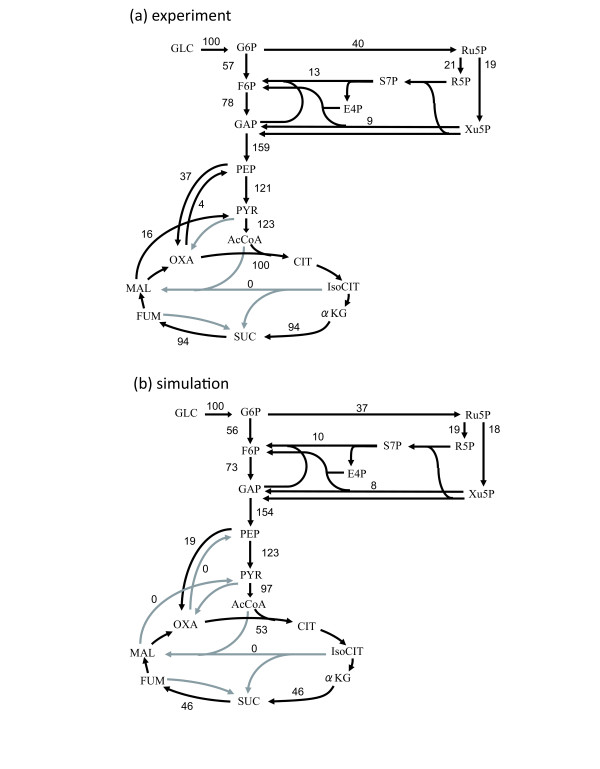
**Metabolic flux profiles of *C. glutamicum *in the exponential growth phase**. (a) The metabolic fluxes obtained by performing ^13^C-tracer experiment [10] and (b) the simulated metabolic fluxes with the same GUR and OUR are presented. The black and gray arrows represent reactions with non-zero and zero fluxes, respectively. Abbreviations are as follows: E4P, erythrose-4-phosphate; 6PG, 6-phospho-d-gluconate; Xu5P, xylulose-5-phosphate; Pyr, pyruvate; F6P, fructose-6-phosphate; GAP, glyceraldehyde-3-phosphate; R5P, ribose-5-phosphate; Ru5P, ribulose-5-phosphate; S7P, sedoheptulose-7-phosphate; G6P, glucose-6-phosphate; LYS, lysine; Suc, succinate; Cit, citrate; IsoCit, Isocitrate; aKG, a-ketoglutarate; MAL, malate; FUM, fumarate; OAA, oxaloacetate.

In order to evaluate the accuracy of the predictions by the FBA simulations in more details, we compared the results of the FBA simulations with the intracellular metabolic flux profile obtained by using a ^13^C-tracer experiment [10]. Here, we referred to the metabolic profile of *C. glutamicum *exponentially grown with glucose as the sole carbon source under aerobic conditions. The GUR and OUR were set to those measured experimentally. As presented in Fig. 2, the predicted flux for the pentose phosphate pathway (PPP) was quite similar to the experimentally obtained flux. However, in the anaplerotic and gluconeogenetic pathways, i.e., phosphoenolpyruvate to/from oxaloacetate, pyruvate to oxaloacetate, and malate to pyruvate, there seemed to be a discrepancy between the predicted and experimentally obtained results. This was because a cycle of metabolic flux in the anaplerotic and gluconeogenetic pathways does not affect the biomass production in the FBA simulation. Therefore, these fluxes were undetermined. However, when we compared the net fluxes from phosphoenolpyruvate/pyruvate to oxaloacetate/malate, which can be uniquely determined by biomass production maximization, the predicted and experimentally obtained fluxes were quite similar (19.5 in the FBA simulation and 18 in the experiment). Additionally, there was a discrepancy in the fluxes of the TCA cycle. The reason for the differences was clear, i.e., it was due to the differences in the precursor demands for the biomass production used in the ^13^C-tracer experiment and our simulation. For example, in our simulation result of aerobic condition, 14.7% of total carbon flux was consumed from acetyl-CoA to synthesis biomass components, such as lipid. In contrast, to obtain fluxes in the ^13^C-tracer experiment shown in Fig. 2, it was assumed that only 7.7% of total carbon flux was used for biomass synthesis from acetyl-CoA. This difference in the precursor demands resulted the discrepancy between simulation and experimental results in the fluxes in TCA cycle. It should be noted that after compensate the difference in the precursor demand, the fluxes in TCA cycle became more similar (data not shown).

### FBA of the metabolic profiles under various oxygen supply conditions

**Figure 3:**
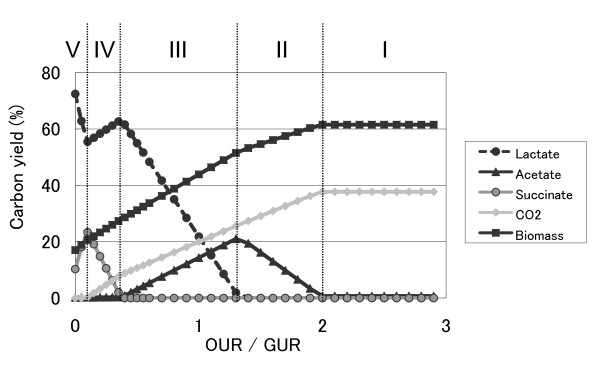
**Result of FBA simulation: changes in the yields of organic acids, biomass, and carbon dioxide by changing the OUR/GUR ratio**.

Since the production yields of biomass, CO_2_, and organic acids agreed well between experimental results and FBA simulations, we further analyzed the changes in the intracellular metabolic profiles on changing the oxygen supply by using FBA simulations of our genome-scale model. In Fig. 3, the changes in the production yields are plotted as a function of OUR/GUR. As shown in the figure, the metabolic profiles can be classified into 5 phases. The schematic representations of the metabolic flux profiles of each phase are presented in additional file 4. Phase I corresponds to the aerobic condition with enough oxygen supply; in this phase, the TCA cycle is activated. On decreasing the oxygen supply, the cells start to produce acetate (phase II). In this phase, the oxygen uptake is not enough to oxidize all the NADH produced when most glucose is converted to CO_2 _in the TCA cycle and the residual glucose is converted to acetate to produce ATP. Further decrease in the oxygen supply results in the production of lactate instead of acetate (phase III). In this phase, the carbon flux to the TCA cycle is almost stopped (except for fluxes to supply metabolites required for the production of biomass such as amino acids). The oxygen uptake is not enough to oxidize all the NADH produced in glycolysis; thus, lactate production is utilized as a mechanism to oxidize NADH in order to maintain the intracellular redox balance. In phase IV, succinate is produced instead of lactate. This is because the capacity of NADH oxidation is greater in the succinate production process than in the lactate production process. Two moles of NADH can be oxidized during the production of 1 mole of succinate from 1 mole of pyruvate, while only 1 mole of NADH can be oxidized during the production of 1 mole of lactate from 1 mole of pyruvate (see the profile (c) and (d) in additional file 4 for details of the metabolic profiles). For this reason, even though the ATP consumption is higher in the anaplerotic pathway than in the lactate production pathway, succinate production is preferred in order to maintain the intracellular redox balance under the micro-aerobic condition in phase IV. Further decrease in the oxygen supply results in an increase in the lactate production again (phase V). In this phase, with an increase in the lactate production, the metabolic flux from malate to pyruvate, which is catalyzed by malic enzyme (ME), is activated and the flux from glycolysis to the PPP decreases (see the profile (e) in additional file 4). Here, the flux for ME is enhanced in order to provide NADPH; the production of NADPH by ME allows a decrease in the flux to PPP, which is major supply source of NADPH in aerobic and microaerobic conditions. The decrease in the PPP flux results in an increase in the carbon fluxes for glycolysis and lactate production, which helps maintain the intracellular redox balance.

As shown in Fig. 1, the changes in the metabolic profiles in the FBA simulations mentioned above agreed well with the changes observed experimentally, except for the succinate production yield in the micro-aerobic and anaerobic conditions (phase IV and phase V). This discrepancy in the succinate production might be due to the differences between the simulation and experiment in the ATP demand for biomass production or in the P/O ratio. As mentioned above, succinate production from pyruvate requires ATP consumption in the anaplerotic pathway; thus, the oxidation of NADH by such succinate production tends to be preferred when the ATP demand for biomass production is small or the P/O ratio is large. In fact, when we changed the ATP demand for biomass production to 70.1 mmol per 1 g biomass, which was 1.7 fold higher than the original coefficient based on the previous report, the predicted production yield of succinate in the micro-aerobic condition showed good agreement with the experimental one. However, this change of coefficient resulted in the decrease of the biomass yield, and a significant discrepancy in the biomass yield between simulation and experimental results arose, for example, it was more than 30% in aerobic condition (OUR/GUR > 2). This result might suggest that the coefficient for ATP in the genome-scale model should be changed depending on OUR.

### Comparison with previously proposed genome-scale model of *C. glutamicum*

The difference between the previously proposed genome-scale model of *C. glutamicum *[25] and our model is summarized in additional file 5. It should be noted that the previous model could not represent the changes that occur in the metabolic profile on altering the oxygen supply. In the FBA simulation using that model, the metabolic profile was not altered by changing the oxygen supply condition. One reason for this discrepancy was the inclusion of inadequate reaction loops in the genome-scale model. For example, that model consists of 2 transport reactions for urea; 1 reaction involves diffusion through the membrane without coupling to any other molecule and the other reaction is the urea-proton symport reaction. In FBA simulations, the combination of 2 such transport reactions results in an arbitrary proton flux from/to inside the cell; for example, the efflux of urea into the medium through the urea-proton symport and the intake of the same amount of urea by diffusion result in proton efflux without any changes in other metabolic balances except that of protons. Of course, the proton efflux can be balanced by proton-ATPase in the cell membrane with the generation of ATP. The genome-scale model reported in Ref. [25] consists several such inadequate reaction loops that allow the arbitrary generation of metabolites such as ATP and NADH; thus, representing the changes in the metabolic profiles by changing the oxygen supply condition is difficult. Furthermore, the model in Ref. [25] lacks the production pathways for lactate and succinate, which also make the representation of the metabolic state in the micro-aerobic condition difficult.

### Analysis of the metabolic profile in the amino acid production phase

**Figure 4:**
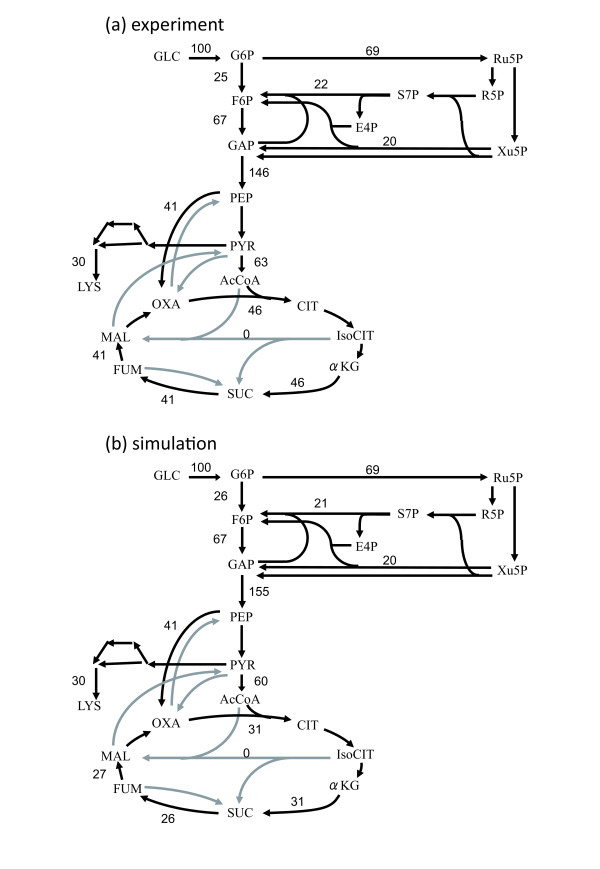
**Metabolic flux profiles of *C. glutamicum *in the lysine production phase**. (a) The metabolic profile obtained by performing the ^3^C-tracer experiment [38] and (b) the simulated metabolic profile obtained by using the same growth rate and by maximizing the lysine production rate are presented.

Since *C. glutamicum *is widely used for the industrial production of various amino acids such as lysine and glutamate, the prediction of metabolic profiles in the production phase of such amino acids is desirable for the improvement of the productivity. Thus, we validated the results of FBA simulation for lysine production by comparing the results with those of ^13^C-tracer experiment presented in Ref. [39]. In order to calculate the lysine production phase, we used lysine production rate instead of biomass production rate as the objective function of FBA to be maximized. The glucose uptake and biomass production rates were fixed at levels observed experimentally. The results revealed that the flux profile predicted by FBA agreed well with an experimentally obtained profile in a previous study, i.e., early production phase with exponential growth (Fig. five in Ref. [39]). These metabolic profiles are presented in Fig. 4. As shown in the figure, the maximal lysine production predicted by the FBA was similar to that obtained in the experiment. Additionally, a higher flux in the PPP in the experimental result (69%) was consistent with that in the FBA simulation (69%), and the net flux in the anaplerotic pathway also exhibited similar values (41% in both the experiment and the simulation). This clear consistency between the simulation and experimental results suggested that the experimentally obtained flux profile corresponds to that with highest lysine productivity under the condition of the observed growth rate. The most significant discrepancy between these 2 flux profiles was observed in the TCA cycle fluxes; this discrepancy was probably due to differences in the pyruvate and acetyl-CoA demands for the biomass production fluxes. Additionally, a discrepancy existed between the simulation result and the experimental result with regard to the use of 2 biosynthesis pathways for diaminopimelate, which is a precursor for lysine biosynthesis [37]. In the model simulation with the maximization of lysine production and fixed biomass production, the diaminopimelate dehydrogenase reaction was preferred; however, the experimental results indicated that both the pathways are active in the lysine production phase [38]. It should also be noted that the flux profile at the late exponential phase presented in Ref. [39] exhibited a larger discrepancy with the predicted flux profile obtained by our genome-scale model. This might be due to the difficulty in predicting a metabolic state at a lower growth rate by using FBA simulation, since there is a negative correlation between the growth rate and the volume of subspace corresponding metabolic state at the growth rate in the feasible flux space of FBA. That is, when the growth rate decreases, the range of possible metabolic states increases. Naturally, the difficulty in predicting the metabolic profile increases with a decrease in the cellular growth rate.

*C. glutamicum *is also widely used for glutamate production. However, the flux profile of the glutamate production phase cannot be represented by using the genome-scale model so far. Glutamate production by *C. glutamicum *can be induced by several triggers such as biotin depletion [40], Tween 40 addition [41], and penicillin addition [42]. After receiving such a trigger, the cells cease growing and then start producing glutamate. Flux analysis using ^13^C-tracer experiments has revealed the changes in the metabolic flux profiles, indicating that a large fraction of carbon derived from glucose is converted to glutamate with the activation of the phosphoenol-pyruvate carboxylase pathway, which provides carbon flux to the TCA cycle [10]. However, the identification of a metabolic state in which glutamate is produced and cell growth is arrested is difficult using analysis by a genome-scale model; this is because the possible range of metabolic states is fairly large and no appropriate objective function exists to predict such a metabolic state. In order to represent the metabolic profile with glutamate production by simulating the genome-scale model, further improvements in the simulation scheme are necessary. For example, it is well known that a decrease in the 2-oxoglutarate dehydrogenase complex activity plays an essential role in the metabolic change in the glutamate production phase [43,44]. Additionally, a recent study suggested the existence of a membrane exporter of glutamate, whose conformation change can be involved in the transition to the glutamate production phase [45]. The inclusion of such regulations of enzymatic activity might be important for the prediction of flux profiles of the glutamate production phase, and should be considered in future studies.

### Analysis of gene deletion for improvement in the production of organic acids

Recently, the production of organic acids by *C. glutamicum *under oxygen deprivation conditions was proposed for increasing the productivity [6]. For the realization of this process, genetic engineering of *C. glutamicum *to improve organic acid productivity is desirable. Using FBA of our *C. glutamicum *metabolic model, we determined candidate genes for target deletion to improve the organic acid productivity under oxygen deprivation conditions. Table 3 shows the candidate pathways whose disruption by gene deletion resulted in an increased production of lactate or succinate. The FBA simulations with biomass production rate as the objective function were performed under the condition of OUR/GUR = 0.1, in which the production rates of lactate and succinate are relatively high (See Fig. 3). For lactate overproduction, disruption of the succinate production pathway was important. Furthermore, the FBA simulation suggested that the disruption of reactions concerned with oxidative phosphorylation increased the lactate production rate. This was because of an increase in the demand of NADH oxidation caused by the disruption of oxidative phosphorylation, which resulted in an increase in the lactate production to oxidize NADH. Additionally, disrupting some reactions in the PPP resulted in an increase in the lactate production, which was due to decrease of NADPH synthesis in the PPP. Then, in order to compensate for NADPH production, which is required for cell growth, the reaction from malate to pyruvate, catalyzed by ME, was activated. The result of the FBA simulation indicated that the increased pyruvate was in turn converted to lactate. For succinate overproduction, disrupting only the lactate production pathway was effective in increasing the production rate of succinate. This result was natural since a large portion of the carbon flux is converted to lactate, as shown in the metabolic profile of the wild type. We further performed multiple gene deletion analysis to screen a set of genes to increase the lactate and succinate production rates; however, no further significant increase in the lactate and succinate production rates was observed (data not shown).

**Table 3:** Candidate reactions whose disruption increases the lactate or succinate production flux predicted by FBA simulations.

Reaction disabled by gene deletion	Lactate production flux (mmol/gDW/h)	Growth rate (1/h)
(Wild type)	3.33 (1.00)	9.54 × 10^-2 ^(1.00)
ADP + Pi + 4H [e] → ATP + H_2_O + H	5.13 (1.54)	8.50 × 10^-2 ^(0.89)
		
R5P + Xu5P ↔ S7P + GAP	5.02 (1.51)	9.06 × 10^-2 ^(0.95)
MAL ↔ FUM + H_2_O	4.99 (1.50)	8.97 × 10^-2 ^(0.94)
G6P + NADP → 6PGL + NADPH + H	4.99 (1.50)	9.06 × 10^-2 ^(0.95)

Reaction disabled by gene deletion	Succinate production flux (mmol/gDW/h)	Growth rate (1/h)

(Wild type)	1.05 (1.00)	9.54 × 10^-2 ^(1.00)
NADH + PYR + H ↔ LAC + NAD	2.24 (2.13)	8.11 × 10^-2 ^(0.85)

Lactate and succinate production fluxes and growth rate of strains in which a reaction is disabled by gene deletion are presented. The production fluxes and growth rate were calculated with the parameters GUR = 0.3 mmol/gDW/h and OUR = 0.03 mmol/gDW/h. Values in parenthesis represent fold change compared with those of wild type. Abbreviations are as follows: H [e], extracellular proton; MAL, malate; FUM, fumarate; G6P, glucose-6-phosphate; 6-PGL, 6-phospho-D-glucono-1,5-lactone; R5P, ribose-5-phosphate; Xu5P, xylulose-5-phosphate; S7P, sedoheptulose-7-phosphate; GAP, glyceraldehyde-3-phosphate; PYR, pyruvate; LAC, l-lactate.

## Conclusion

In this study, we developed a genome-scale metabolic model of *C. glutamicum*, which is commercially important for the production of amino acids and useful chemicals. This model includes hundreds of metabolites and reactions among them, and also includes that the hypothetical reaction representing synthesis of biomass to calculate the requirement of metabolites for cell growth. It should be stressed that, this model is not just a collection of all the public available data about the metabolic reaction of C. glutamicum. Instead, we constructed the model by adding and deleting metabolic reactions to/from those on the databases, to make this model available for the flux balance analysis. For example, based on some literatures, we added some reactions to the metabolic model to fill gaps in the network to allow biomass formation. Also, we removed some reactions to avoid the formation of inadequate loop reactions, which makes impossible to calculate balance of metabolic fluxes due to the arbitrary generation of metabolites by this loop reaction as discussed above. Only after fixing these problems, we could use the metabolic model for FBA and predict the metabolic profiles. Using the genome-scale model, we performed FBA to understand the characteristics of the metabolic network and to identify candidates of the target metabolic pathways that can be manipulated to improve organic acid production by *C. glutamicum*. The results revealed that the FBA agreed well with the experimental results; this suggests that when the cells grow exponentially, the metabolic profiles can be predicted by our genome-scale model with maximization of the biomass production rate. We also performed simulations to predict the metabolic profiles in amino acid production phases, and succeeded in representing the metabolic profiles in the lysine production phase. However, the glutamate production phase, in which the cells stop growing, could not be represented by the genome-scale model. Further improvements in the model, such as inclusion of some gene regulatory machinery, should be considered. Furthermore, we performed *in silico *screening to identify candidates of the target metabolic pathways that can be manipulated to improve organic acid production by *C. glutamicum*. Our results revealed that the disruption of H^+^-ATPase activity is the most effective in improving lactate production under oxygen deprivation conditions. In fact, recent studies showed that the disruption of H^+^-ATPase results in significant changes of metabolic flux profiles in *C. glutamicum *[46], although the flux profiles under oxygen deprivation have not yet been investigated. The experimental verification of this *in silico *screening remains as future works. We expect that further extensive studies using our genome-scale model with experimental verifications will enable us to understand in detail the characteristics of the metabolic networks of *C. glutamicum*.

## Competing interests

The authors declare that they have no competing interests.

## Authors' contributions

YS and MS performed the model construction and simulations. NS performed the culture experiments. CF participated in the design of the study and drafted the manuscript. TH rewrote the manuscript. HS conceived and supervised the study. All authors revised and approved the final manuscript.

## Supplementary Material

Additional file 1 **List of metabolic reactions in the *C. glutamicum *genome-scale model**. The sheet "reactions" contains all the reactions in the *C. glutamicum *genome-scale model categorized by their metabolic pathway and the sheet "abbreviated metabolites" enlists the abbreviations of the metabolites. Click here for file

Additional file 2 **Growth and organic acid production by C. glutamicum under the anaerobic condition (experiment 1)**. The culture was initiated under aerobic conditions with air aeration and a high agitation speed, and 9 h after inoculation (indicated by a black arrow) the culture conditions were changed to no aeration and gentle agitation (100 rpm). The GUR, OUR, and production rates of organic acids, biomass, and carbon dioxide were calculated using the data obtained from 9 to 15 h after inoculation. Click here for file

Additional file 3 **Growth and organic acid production by *C. glutamicum *under microaerobic conditions (experiment 2)**. The culture was initiated under aerobic conditions with air aeration and a high agitation speed, and 9 h after inoculation (indicated by a black arrow) the aeration rate was changed to 0.5 vvm and the OUR was maintained at a constant value (0.5 mmol/gDW/h) by changing the agitation speed. The GUR, OUR, and production rates of organic acids, biomass, and carbon dioxide were calculated using the data obtained 9 to 15 h after inoculation. Click here for file

Additional file 4 **Metabolic flux profiles of *C. glutamicum *in the different phases**. Figures (a) ~(e) show the schematic representations of the metabolic profiles in phases I ~ V, respectively. The thickness of the green arrows roughly corresponds to the metabolic fluxes; the gray arrows represent reactions with zero flux. The following abbreviations are used: Re_Mq, reduced form of menaquinone; Ox_Mq, oxidized form of menaquinone. Click here for file

Additional file 5 **Summary of differences between the genome-scale models of C. glutamicum in Ref.**[25]** and present study**. The essential differences between the previous study [25] and our study are presented. Click here for file
