# Supplementary figures and images for: Development and experimental verification of a genome-scale metabolic model for Corynebacterium glutamicum
Source: Microb Cell Fact. 2009 Aug 3;8:43. doi: 10.1186/1475-2859-8-43 (PMC2728707; doi:10.1186/1475-2859-8-43)

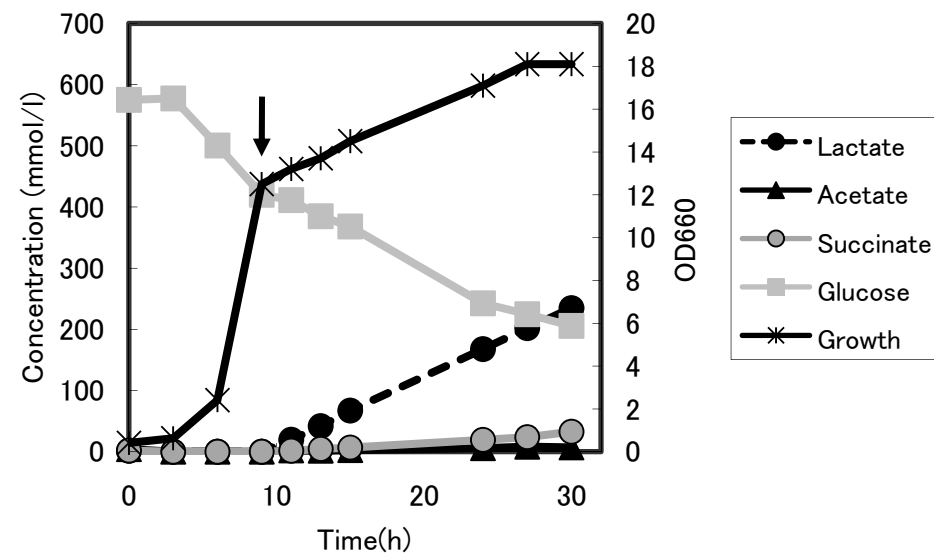

Supplement: Additional file 2 — Growth and organic acid production by C. glutamicum under the anaerobic condition (experiment 1). The culture was initiated under aerobic conditions with air aeration and a high agitation speed, and 9 h after inoculation (indicated by a black arrow) the culture conditions were changed to no aeration and gentle agitation (100 rpm). The GUR, OUR, and production rates of organic acids, biomass, and carbon dioxide were calculated using the data obtained from 9 to 15 h after inoculation. [file 1475-2859-8-43-S2.pdf]

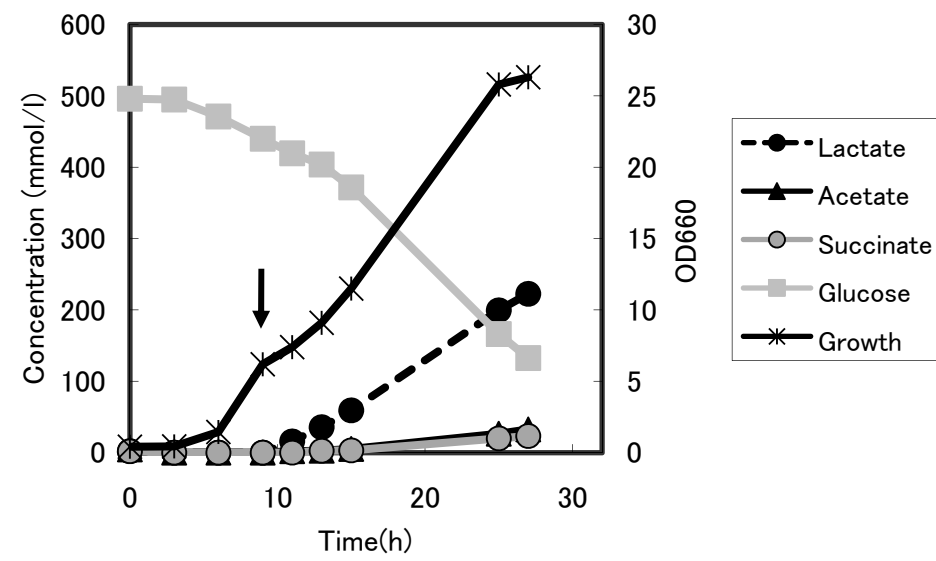

Supplement: Additional file 3 — Growth and organic acid production by C. glutamicum under microaerobic conditions (experiment 2). The culture was initiated under aerobic conditions with air aeration and a high agitation speed, and 9 h after inoculation (indicated by a black arrow) the aeration rate was changed to 0.5 vvm and the OUR was maintained at a constant value (0.5 mmol/gDW/h) by changing the agitation speed. The GUR, OUR, and production rates of organic acids, biomass, and carbon dioxide were calculated using the data obtained 9 to 15 h after inoculation. [file 1475-2859-8-43-S3.pdf]

(a)

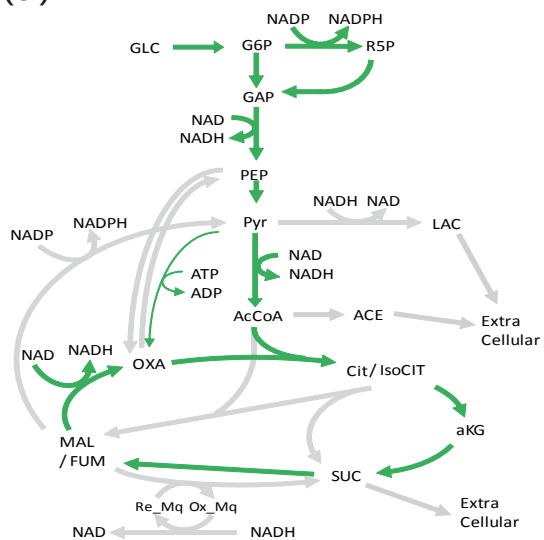

(b)

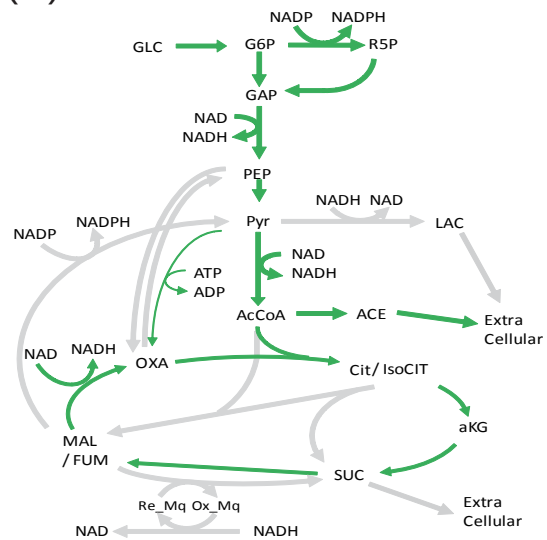

(c)

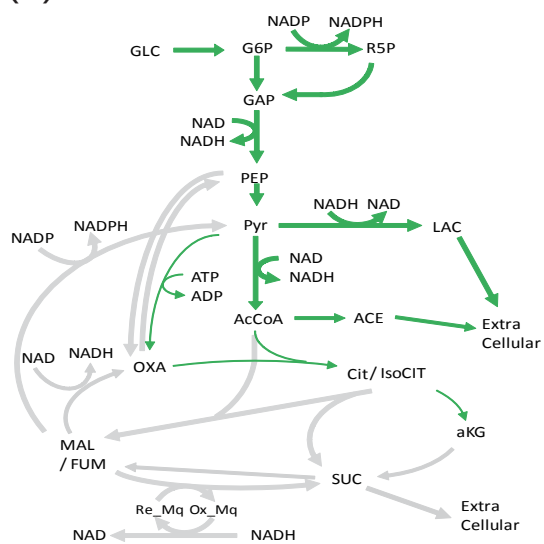

(d)

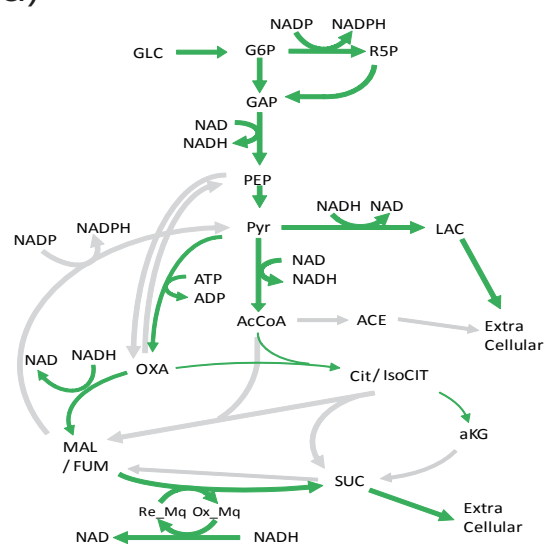

(e)

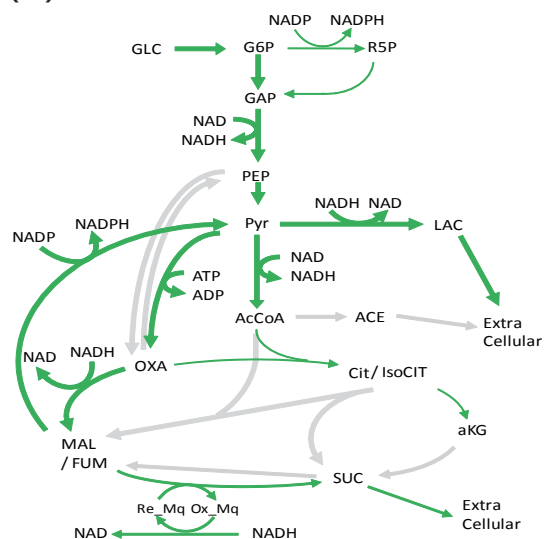

Supplement: Additional file 4 — Metabolic flux profiles of C. glutamicum in the different phases. Figures (a) ~(e) show the schematic representations of the metabolic profiles in phases I ~ V, respectively. The thickness of the green arrows roughly corresponds to the metabolic fluxes; the gray arrows represent reactions with zero flux. The following abbreviations are used: Re_Mq, reduced form of menaquinone; Ox_Mq, oxidized form of menaquinone. [file 1475-2859-8-43-S4.pdf]
