# Supplementary material for: Development and experimental verification of a genome-scale metabolic model for Corynebacterium glutamicum
Source: Microb Cell Fact. 2009 Aug 3;8:43. doi: 10.1186/1475-2859-8-43 (PMC2728707; doi:10.1186/1475-2859-8-43)
Supplement: Additional file 5 — Summary of differences between the genome-scale models of C. glutamicum in Ref.[25] and present study. The essential differences between the previous study [25] and our study are presented. [file 1475-2859-8-43-S5.pdf]

# Additional file 5

| | Previous model [25] | Our model |
|---|---|---|
| **Number of reactions in genome scale model** | 446 | 502 |
| **Number of metabolites in genome-scale model** | 411 | 423 |
| **Inadequate reaction loop that allows arbitrary generation of metabolites** | Include | Not include |
| **Representation of metabolic state in micro-aerobic condition** | Difficult | Possible |
